# Estimating 3D ground reaction forces in running using three inertial measurement units

**DOI:** 10.3389/fspor.2023.1176466

**Published:** 2023-05-15

**Authors:** Bouke L. Scheltinga, Joost N. Kok, Jaap H. Buurke, Jasper Reenalda

**Affiliations:** ^1^ Biomedical Signals and Systems, Faculty of Electrical Engineering, Mathematics and Computer Science (EEMCS), University of Twente, Enschede, Netherlands; ^2^ Faculty of Electrical Engineering, Mathematics and Computer Science (EEMCS), University of Twente, Enschede, Netherlands; ^3^ Roessingh Research and Development, Enschede, Netherlands

**Keywords:** running, ground reaction force, estimation, machine learning, ensemble model

## Abstract

To understand the mechanisms causing running injuries, it is crucial to get insights into biomechanical loading in the runners' environment. Ground reaction forces (GRFs) describe the external forces on the body during running, however, measuring these forces is usually only possible in a gait laboratory. Previous studies show that it is possible to use inertial measurement units (IMUs) to estimate vertical forces, however, forces in anterior-posterior direction play an important role in the push-off. Furthermore, to perform an inverse dynamics approach, for modelling tissue specific loads, 3D GRFs are needed as input. Therefore, the goal of this work was to estimate 3D GRFs using three inertial measurement units. Twelve rear foot strike runners did nine trials at three different velocities (10, 12 and 14 km/h) and three stride frequencies (preferred and preferred ± 10%) on an instrumented treadmill. Then, data from IMUs placed on the pelvis and lower legs were used as input for artificial neural networks (ANNs) to estimate 3D GRFs. Additionally, estimated vertical GRF from a physical model was used as input to create a hybrid machine learning model. Using different splits in validation and training data, different ANNs were fitted and assembled into an ensemble model. Leave-one-subject-out cross-validation was used to validate the models. Performance of the machine learning, hybrid machine learning and a physical model were compared. The estimated vs. measured GRF for the hybrid model had a RMSE normalized over the full range of values of 10.8, 7.8 and 6.8% and a Pearson correlation coefficient of 0.58, 0.91, 0.97 for the mediolateral direction, posterior-anterior and vertical direction respectively. Performance for the three compared models was similar. The ensemble models showed higher model accuracy compared to the ensemble-members. This study is the first to estimate 3D GRF during continuous running from IMUs and shows that it is possible to estimate GRF in posterior-anterior and vertical direction, making it possible to estimate these forces in the outdoor setting. This step towards quantification of biomechanical load in the runners' environment is helpful to gain a better understanding of the development of running injuries.

## 1. Introduction

Although running is a very healthy activity (1), it causes many injuries worldwide (2). With an average recovery time of 58 days (3), musculoskeletal running injuries form a barrier to continue running. In fact, injuries are the main reason people completely quit running (4, 5). Currently, the overall evidence for the effectiveness of interventions to prevent lower-limb injuries in running is very limited (6). This can be explained by the fact that the aetiology of running-related injuries is not established as it is complex and multifactorial (7). To get a better understanding of the development of injuries, research is needed that analyses the change prior to an injury and change in the (a) amount of participation, (b) load distribution, (c) magnitude of the load or (d) load capacity (8). Thus, it is important to quantify biomechanical load longitudinally in research on running injuries (9).

Ground reaction force (GRF) is the force that the runner exerts on the ground every single step in vertical, anterior-posterior (AP) and mediolateral (ML) directions. With this force as input, combined with an inverse dynamics approach, structure-specific body loads can be calculated, such as tibial bone load, joint contact force or Achilles tendon force (10). GRF itself provides a generic indication of biomechanical load for the musculoskeletal system as a whole (11). Besides monitoring training load, GRF can also be used to calculate joint powers. The latter is useful, for example, to investigate the effect of different footwear properties (12).

The gold standard to measure GRFs in running is using instrumented treadmills or force plates embedded in the floor of a laboratory. However, these lab-based methods are different from the runners' natural outside environment, and it is shown that there are significant differences in kinematics between outdoor running and running on a treadmill (13). As such, it can be assumed that kinetics will also be different in outdoor running. Furthermore, when performing a longitudinal study to monitor training loads, it is not realistic to have the participants running on an instrumented treadmill. This stresses the importance of a GRF estimation outside the laboratory environment.

Inertial sensor technology makes it possible to move outside of the lab into the runner's environment. For example, it is already shown that it is possible to show fatiguing effects on running kinematics during a marathon using inertial measurement units (IMUs) (14). IMUs can also be used to estimate the peak in vertical GRF, as done using a statistical method (15, 16). As one of the sensors within the IMU is an accelerometer, acceleration can easily be used to calculate forces using Newton's second law of motion by multiplying acceleration with body mass. This was applied by Wundersitz et al. (2014), and it was concluded that accelerometers could asses differences in peak impact forces over time (17). More recently, a complete vertical GRF waveform was constructed from three IMUs using a general physical model, achieving an average root mean squared error (RMSE) of 0.18 body weight (BW) (18). Using a mass-spring-damper model, it was even possible to estimate vertical GRF waveforms during running with data from a single IMU (19).

With the increase in availability and usability of wearable sensors, the ease of collecting data has increased. As more data are available, it opens up the opportunity to use machine learning, which requires many observations (20). In 2018, an artificial neural network was used to estimate the vertical GRF waveform in running using 3 IMUs, reaching a mean RMSE <0.27BW (21). To our knowledge, the best estimates for GRF waveforms are achieved using bidirectional long short-term memory (LSTM) network, with a mean RMSE of 0.16BW, for GRF perpendicular to the ground surface, on various slopes (22). However, the latter study calculated the RMSE over multiple strides, including the flight phase. As the GRF during flight phase is easy to predict (0N), this lowers the RMSE value compared to studies that used GRF over stance phase only.

So far, work in estimating GRF in continuous running using IMUs is all limited to a single dimension. However, human movement is in three-dimensions, meaning that there are also forces in three directions. AP forces are actually needed together with the vertical forces to calculate the provided power by the limbs in the sagittal plane (23). Even more, to use inverse dynamics in 3D, GRFs in all dimensions are needed. 3D GRF estimation is already done for walking gait (24–26) or for running tasks, such as acceleration or change of direction (27). However, 3D GRF estimation during continuous running using IMUs is currently lacking in the literature.

Assembling multiple models into an ensemble can result in a better performance than single models (28), for example, as shown in a study on estimating ankle moment (29). This approach is rarely used in the field of biomechanics and not applied for the estimation of 3D GRF. An ensemble model can be constructed by taking the average prediction from ensemble-members. This approach has the potential to improve the accuracy of the model by leveraging the strengths of each individual model, as well as by mitigating the effects of overfitting. Additionally, combining different models can provide a more robust model that is less sensitive to variations in the training data, leading to better generalization on unseen data. To our knowledge, this approach is uncommon in biomechanics. However, it could boost machine learning model performance to estimate biomechanical parameters like GRF.

Machine learning models to estimate GRF can be either generalized or personalized. It is evident that personalized machine learning models will result in much better model performance, as shown previously (21, 22). Personal models can be useful for research purposes, in which biomechanical load data of a small number of subjects will be tracked longitudinally, after creation of personal machine learning models. However, creating these personal models is time consuming, especially if more subjects are included, and requires the availability of a (3D) instrumented treadmill. Creating generic models is more challenging, as they should be able to handle between-subject variability in the data (20). The upside is that these models can be used “out of the box”. Once a generic model is created, no specific model training is needed to estimate 3D GRF for new subjects. Therefore, this work focussed on generic models only.

As physics-based techniques and machine learning have both been used to estimate vertical GRF, it opens up the opportunity to apply a so-called hybrid model. With a hybrid model, the domain knowledge of the physics-based model is combined with a machine learning model, and this combination can lead to improved model performance (27). For example, it is applied with success in chemical engineering, where physical conservation laws were applied and corrected by an artificial neural network that estimated the error of the physical laws (30). Another method could be to enrich the input space of the model with a physical estimate. With a hybrid model, the physical explanatory power can be used while the benefits of fitting with machine learning are utilized.

This study aims to develop a method to estimate 3D GRF waveforms during running using data from three IMUs on the human body. This model could be used to estimate GRF in the runner's environment to get insights in biomechanical loading, in the absence of force measurement. An ensemble artificial neural network (ANN) will be used to create generic models based on data for running at different velocities and stride frequencies. Additionally, an ensemble hybrid ANN will be used to combine the exploratory power of a physical model with the fitting performance of machine learning. It is hypothesized that this hybrid model will outperform the physical and machine learning-based approaches published in other research. An accurate GRF estimation for monitoring biomechanical load in the runners environment could help providing future insights in aetiology for running injuries.

## 2. Materials and methods

Sixteen runners participated in the study after signing informed consent. Inclusion criteria consisted of (1) running a minimum of 15 km/week for the last six months, (2) running with a heel strike, (3) being able to run 14 km/h for 5 min and (4) no major injuries in the past six months. A major injury was defined as an injury that caused a runner to shorten runs or skip runs because of the injury. Participants were recruited *via* local athletics and triathlon associations. The ethics committees (CCMO Arnhem/Nijmegen and the University of Twente) approved the protocol.

### 2.1. Measurement setup

Participants were equipped with eight IMUs following the lower body configuration from the manufacturer (Xsens MTx, Xsens Technologies, Enschede, The Netherlands) with a sampling frequency of 240 Hz. As part of a larger study, a total of eight sensors were placed, on the feet, (proximal) tibias, thighs, pelvis, and trunk. Sensors were placed using double-sided tape and covered with additional tape. Leg sleeves were pulled over the sensors on the tibias. A sensor-to-segment calibration was performed according to the sensor manufacturer's instructions. GRF data were collected with the 3D force plate instrumented dual-belt treadmill (Y-mill, Motek Medical, Amsterdam, The Netherlands), with a sampling frequency of 2,048 Hz.

### 2.2. Running protocol

Before the first running trial, a static trial was performed on the treadmill, which was used to obtain the subjects' body mass. Subsequently, the participants ran in random order at 10, 12 and 14 km/h (2.8, 3.3 and 3.9 m/s) at a preferred, high, and low stride frequency. The high and low stride frequencies were determined by the preferred stride frequency ± 10%. The stride frequencies were imposed using a metronome, indicating each foot strike. Subjects ran each trial for 90 s. Each trial started and finished with three vertical jumps to synchronize the IMUs and treadmill (see Data processing). There was a three-minute break between each trial to prevent the participants from fatiguing.

### 2.3. Data processing

The data from the treadmill was filtered using a sixth order, zero-phase shift low pass Butterworth filter with a cut-off frequency of 30 Hz. Then the data were down sampled to 240 Hz, to match the IMU system. The IMU output used was 3D sensor free acceleration, this gravity subtracted acceleration in the global frame, was used in this work. The cross-correlation between the sum of the sensor free acceleration along the axis of both tibias and vertical GRF was used to find the temporal offset between the two systems. From the middle of each trial, 40 strides were taken. Flight-phase was detected and labelled if the measured vertical GRF was <25N for longer than 0.05s, it was labelled as stance-phase if the measured GRF (mGRF) was >25N for longer than 0.05s.

### 2.4. Model structures

**Figure 1:**
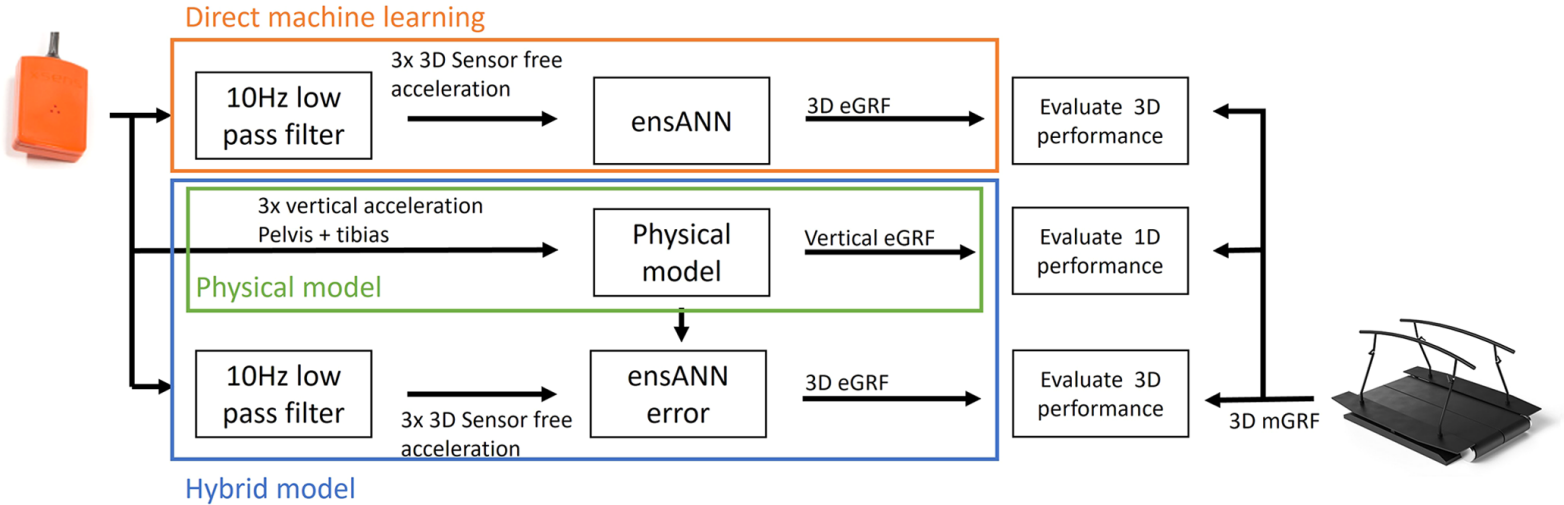
Overview of the different models. A ensemble artificial neural network (ensANN) was used to estimate 3-dimensinal ground reaction force (3D eGRF directly). Also, a hybrid model was created by adding the physical estimate as additional input for the ensANN. The estimates of the models were then compared with measured GRF (mGRF) to evaluate the performance.

In this study, a direct machine learning model (“direct”) and hybrid machine learning model (“hybrid”) were trained, tested, and compared to a physical model. The direct and hybrid model are comparable in structure, the main difference is that the hybrid model uses the physical estimate as input, in addition to the sensor acceleration data (Figure 1).

For the physical estimate, a previously developed model based on Newton's second law was used to estimate the vertical GRF from IMU data from the pelvis and both tibias (18). The vertical GRF is estimated as:

$$eGRF=(m_{b}\times g)+\overset{3}{\underset{i}{\sum }}m_{b}\times WF_{i}\times (a_{z,i}),$$

with body mass *m_b_*, gravitational acceleration *g*, sensor number *i*, weight factor *WF_i_* and sensor free acceleration in the vertical direction $a_{z}$. The weight factor corresponding to the pelvis data was set to 0.55. The weight factor corresponding to the tibias was set to 0.23. The sensor free acceleration in the vertical direction was filtered for the pelvis using a 2nd order bidirectional Butterworth filter with a cut-off frequency at 5.97 Hz. The tibia sensor free acceleration in vertical direction was filtered using a 1st order bidirectional Butterworth filter with a cut-off frequency at 8.74 Hz. The weight factors and cut-off frequencies are taken from (18), where a model optimization resulted in this set of parameters to estimate vertical GRF. As there are no physical models for the mediolateral and posterior-anterior direction in running, only the vertical direction is used as physical input for the hybrid model.

### 2.5. Machine learning model architecture

Both the direct and hybrid models use the same ANN architecture. The sensor-free acceleration data were filtered using a 3rd order bidirectional Butterworth low-pass filter with a cut-off frequency at 10 Hz. The used ANNs had 2 layers with 100 neurons and ReLU activation function. Mean squared error was used as the loss function, and adaptive moment estimation (Adam) was used as the update rule (31). Models were trained for 1,000 epochs, with a batch size of 250. An early stopping criterium was implemented as the validation loss did not decrease for more than 100 epochs. In that case, the model with the lowest validation loss was used. All models were implemented in python 3.8 using the TensorFlow backend (32).

### 2.6. Model testing and ensemble

**Figure 2:**
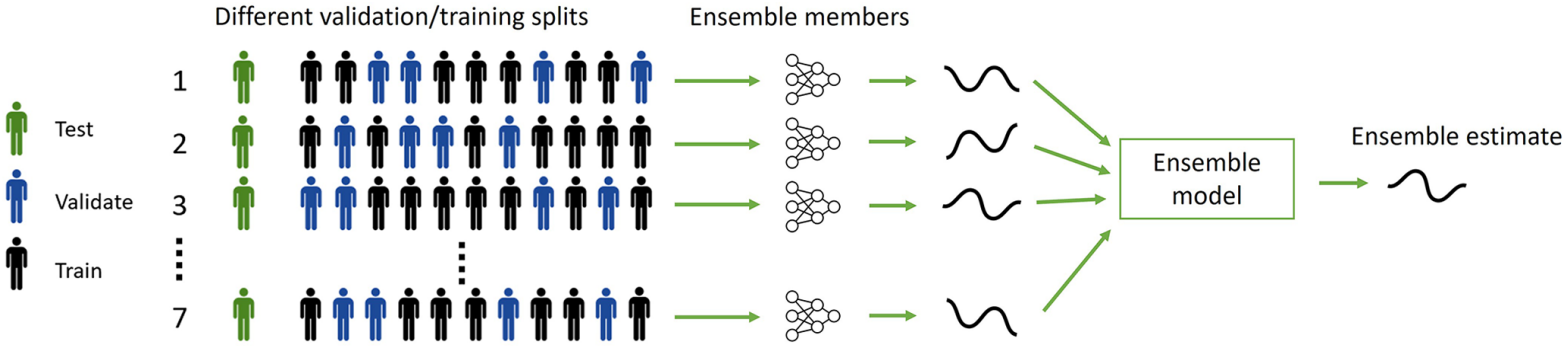
The leave-one-subject-out cross-validation process to create an ensemble model with 7 random validation-test splits per subject, with four subjects for validation and 7 for training. Every combination is used as an ensemble-member and combined in the ensemble model. After 7 models were trained for one subject, the test subject was changed and repeated until seven different models were fitted for every test to have a leave-one-subject-out cross validation.

Data of one subject was left out from the data set to train and validate the models on the other subjects. Seven different models were trained with randomly created splits with four subjects for validation and seven subjects for training (Figure 2). Data from all velocities and stride frequencies were used. Then, the average estimated GRF (eGRF) over these seven models was calculated for the left-out-subject to get the ensemble eGRF. This process was repeated, and every subject was left out. This leave-one-subject-out cross-validation was used to test how well the models work for the data of subjects that the model was never trained or validated for (33).

### 2.7. Model evaluation

To evaluate the models in terms of accuracy, the root mean square error (RMSE) of the normalized GRF expressed in bodyweight and Pearson correlation coefficient were calculated between the eGRF and mGRF for all three directions. To compare the estimations between the different directions, the relative RMSE (rRMSE) was calculated as

$$rRMSE=\;\frac{RMSE}{\max(mGRF)-\min(mGRF)}*100,$$

where the RMSE is corrected for the full range of the mGRF waveform for that specific trial. Besides the RMSE, also the absolute active peak error was calculated as the percentual difference between the estimated and measured peak in the vertical direction. To evaluate the models in terms of precision, the Pearson correlation coefficients between the estimated and mGRF waveforms were calculated. The mentioned error measures were also calculated for the physical model to compare performance between the physical and machine learning methods.

A dependent t-test for paired samples was performed between the different models on the model evaluation parameters to test the significance of the difference between model performance.

## 3. Results

Data of 12 subjects (4 female, 8 male, 31.6 ± 9.0 years, 1.78 ± 0.11 m, 73.7 ± 17.5 kg). was included, while data from 4 subjects had to be excluded (1 × wrong calibration, 1 × not finished protocol, 2 × not heel strikers). For one subject, two trials were missing, meaning that a total of 3,520 ((10*3*3*40)−2 × 40) strides were used to train, validate and test the models.

**Figure 3:**
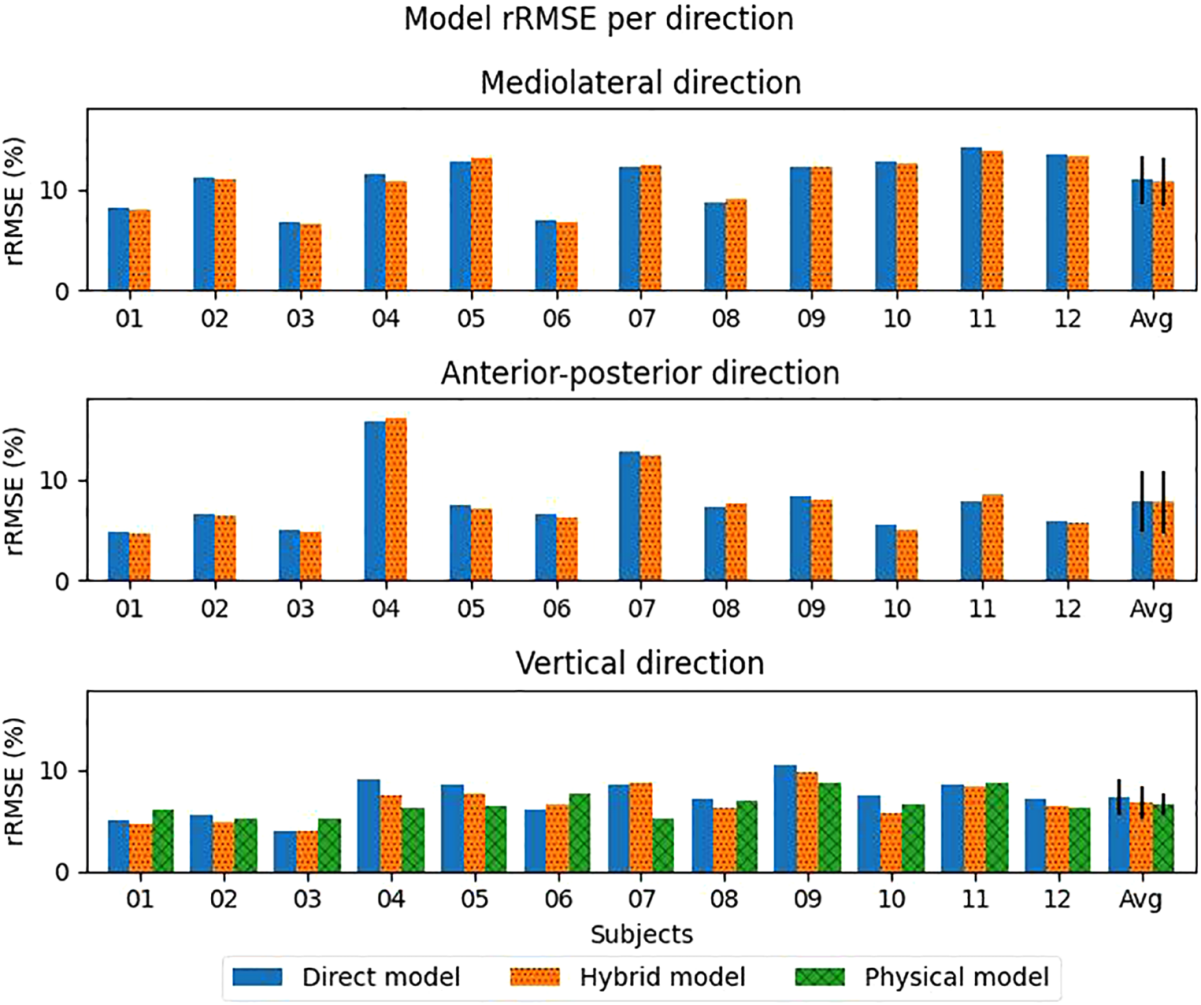
Relative root mean squared error (rRMSE) for each axis per model per subject. The exact data can be seen in Supplementary material Table S1. The error bars indicate the standard deviation.

Leave one-subject-out cross-validation showed that the rRMSE was lowest in vertical direction for all used models (Table 1). Although the physical model had the lowest rRMSE (6.6%), it was not significantly lower than the direct and hybrid models. The hybrid model was significantly better than the direct model in vertical direction, with an rRMSE of respectively 7.0 and 7.6%. In ML and AP direction, the rRMSE values were higher (>8.2%) compared to the vertical direction, meaning a less accurate estimate. The variation between subjects and models is shown in Figure 3.

**Table 1:** Model performance for the different models in the different directions. With root mean squared error (RMSE) in bodyweight (BW), relative RMSE (rRMSE) as percentage.

Direction	Model	RMSE (BW)	rRMSE (%)	Pearson's r	Peak error (%)
Mediolateral	Direct	0.05 ± 0.01	10.9 ± 2.5	0.57 ± 0.21	–
	Hybrid	0.05 ± 0.01	10.8 ± 2.5	0.58 ± 0.22	–
Anterior-posterior	Direct	0.08 ± 0.03	7.8 ± 3.1	0.91 ± 0.09	–
	Hybrid	0.07 ± 0.03	7.8 ± 3.2	0.91 ± 0.10	–
Vertical	Direct	0.19 ± 0.04*	7.3 ± 1.8*	0.97 ± 0.02*^+^	4.21 ± 1.36
	Hybrid	0.18 ± 0.04*	6.8 ± 1.7*	0.97 ± 0.01*	4.09 ± 1.35
	Physical	0.18 ± 0.03	6.6 ± 1.2	0.98 ± 0.01^+^	3.61 ± 1.80

* The denotes a significant difference between the direct and hybrid model with alpha < 0.05.

^+^ The denotes a significant difference between the direct and physical model with alpha < 0.05.

**Figure 4:**
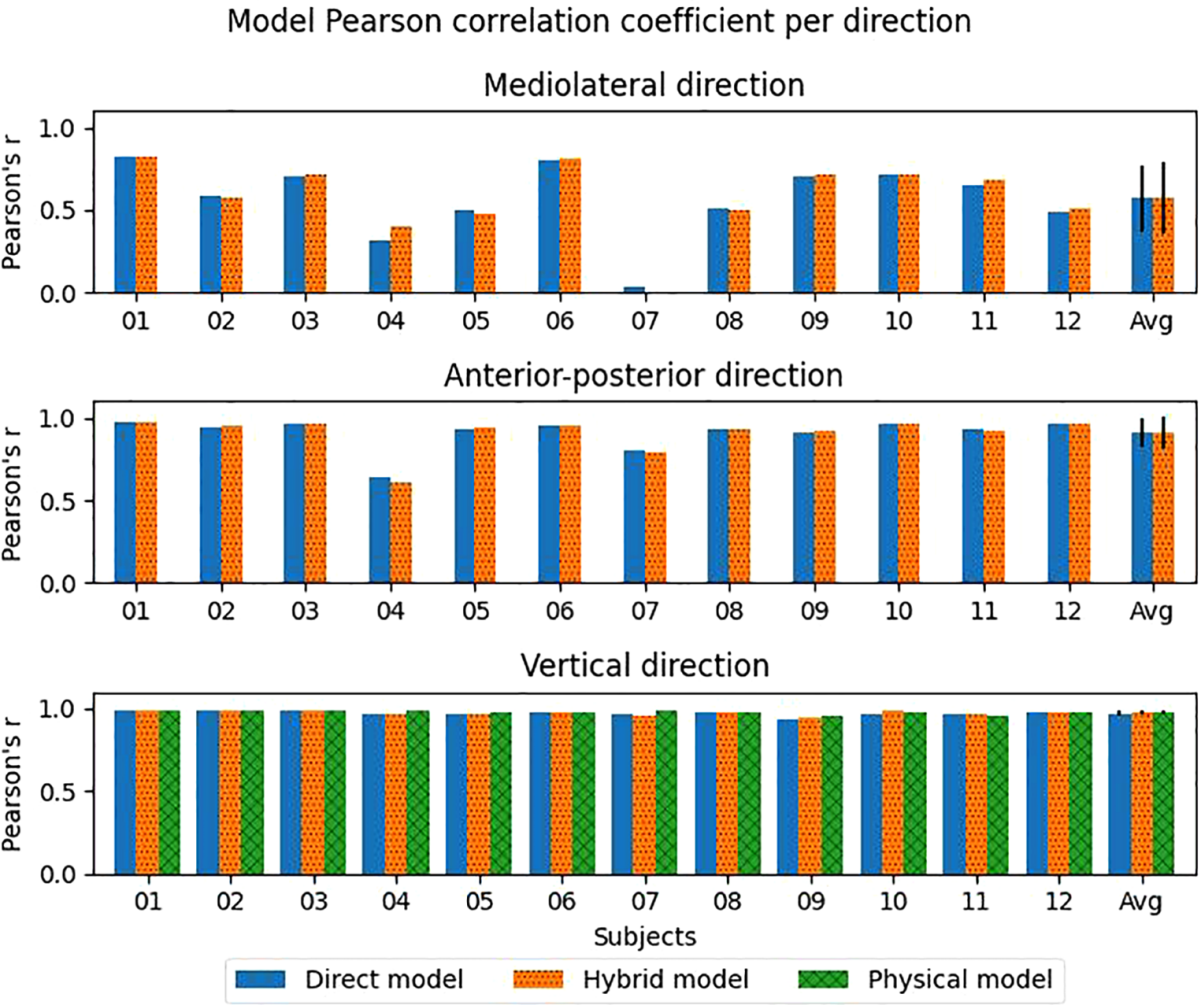
Pearon's r for each axis per model per subject. The exact data can be seen in Supplementary material Table S2. The error bars indicate the standard deviation.

Pearson correlation coefficients between the estimates and measured values were moderate (0.57, 0.58) for the direct and hybrid model in the ML direction. However, a large spread between the subjects was seen (Figure 4). Even a negative correlation coefficient was seen for the hybrid model for subject 07. In the AP and vertical direction, a strong correlation (>0.90) was found on average for both the direct and hybrid model (Table 1).

**Figure 5:**
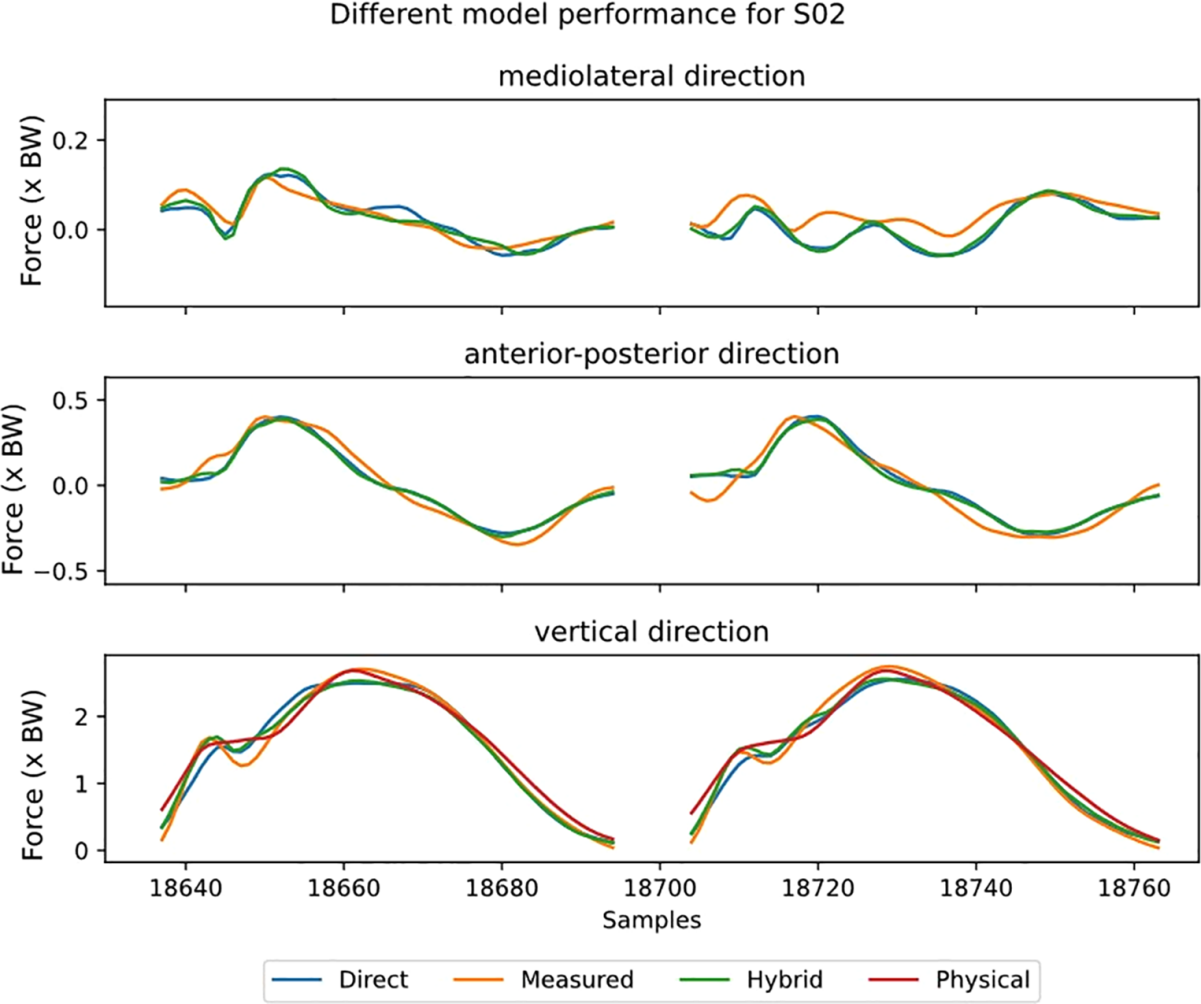
The estimated ground reaction curves for the different axes during stance for all models at 12 km/h preferred stride frequency. With the direct model in blue, hybrid in green, physical in red and reference in orange. Stance phase is replaced by a fixed gap in the data. Note that the y-axis is not the same scale for the different sub-plots.

**Figure 6:**
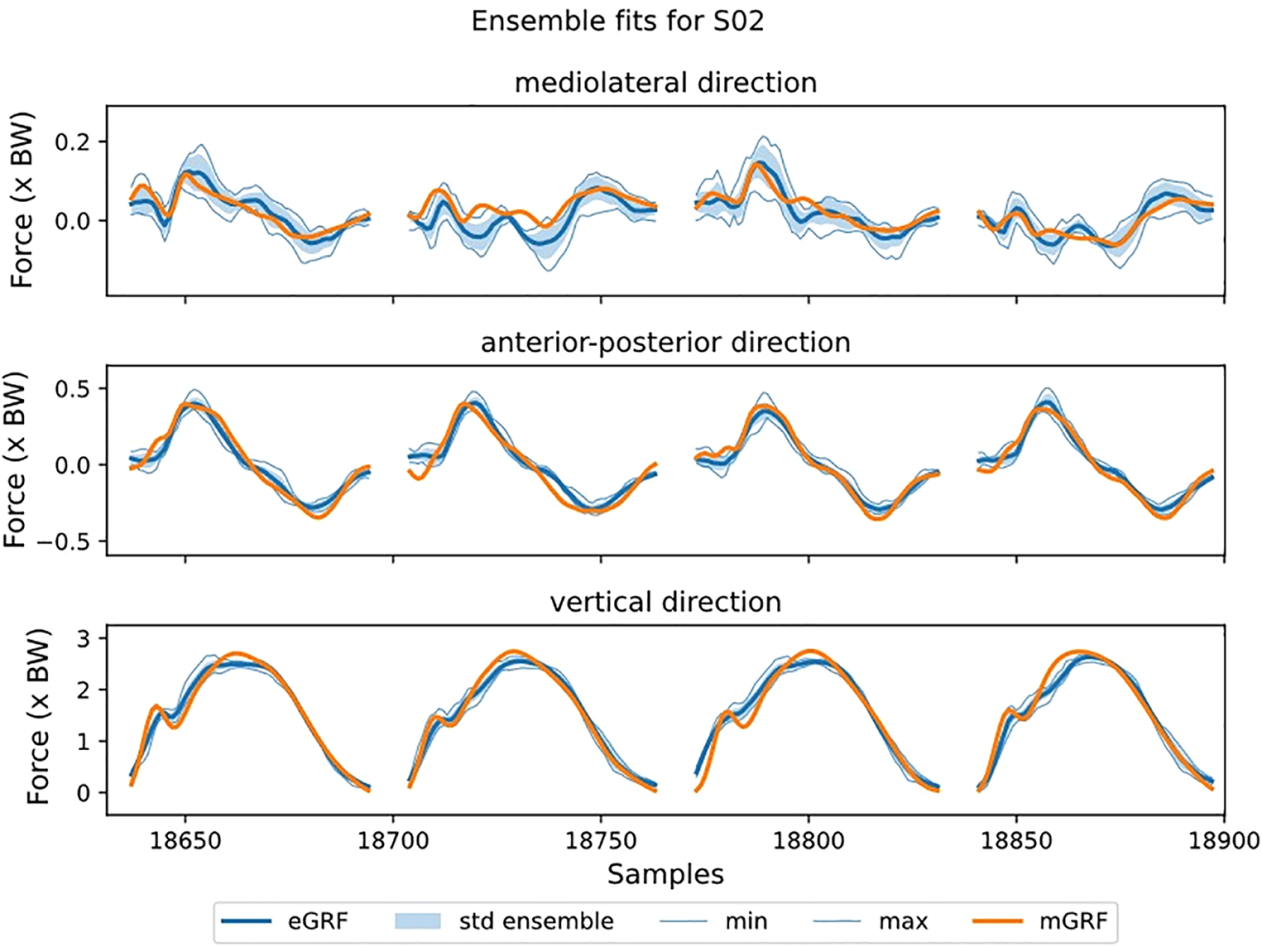
The estimated ground reaction curves for the different axes during stance from the direct model at 12 km/h preferred stride frequency. The shading indicates the average over the ensemble-members plus/minus one standard deviation. Also, the maximum and minimum values are plotted. Stance phase is replaced by a fixed gap in the data. Note that the y-axis is not the same scale for the different sub-plots.

Visual inspection of the GRF waveforms shows that most errors in the estimate are directly after the impact peak (Figure 5). There are only minor differences between the direct and hybrid model for the ML and AP direction. For the ensemble models, also the standard deviation for the ensemble-members (individual results of leave-one-out-subject-validation) is plotted (Figure 6). This standard deviation is typically higher at peak forces. Note also the difference in magnitude of the forces, the ML force is roughly between −0.2 and 0.2 BW, AP force between −0.5 and 0.5 and the vertical force between 0 and 3BW for the shown subject.

The ensemble models outperformed most ensemble-members. For example, for subject 01 in vertical direction, the members had an rRMSE between 4.9 and 6.7%, the ensemble model had an rRMSE of 4.7% (Figure 6). This means that the ensemble model outperforms the best ensemble-member. Furthermore, for 20 out of 36 GRF estimates, the ensemble model had similar or better performance than the best ensemble-member.

## 4. Discussion

Two different ensemble artificial neural networks were developed to predict continuous, 3D GRF in real-time, with the final goal to estimate GRF in the runners' environment. With a rRMSE of 6.8% over all subjects in vertical direction, the hybrid model outperformed the direct model (rRMSE of 7.3%). Also, the relative peak error of the hybrid model was lower than the direct model (4.1 vs. 4.2%). The rRMSE and relative peak error indicate that the hybrid model had a higher accuracy than the direct model in the vertical direction. The precision of the models in the vertical direction was confirmed by a very strong correlation with the mGRF (Pearson's *r* = 0.97). The performance of the hybrid and direct model in vertical direction was comparable with the physical model performance. Model performance was related to the magnitude of the estimated forces, meaning that lower forces, e.g. in ML direction, had higher errors. The findings suggest that, using an ensemble hybrid model, it is possible to estimate GRF in the AP and vertical direction using data from an IMU on the pelvis and one on each tibia. With the current approach, it is not possible, to precisely estimate the ML forces (Pearson's *r* = 0.58).

Although work is done on estimating 3D GRF from IMUs in walking gait (24–26), and in specific running tasks (34), this is the first study that used 3 IMUs with the goal to predict GRF in 3D during continuous running. An earlier study estimated 2D GRF from plantar pressure soles, using a bidirectional LSTM (23). A rRMSE of 8.0% in AP direction and 7.7% in vertical direction, which is slightly higher than the results in this study (7.8% and 6.8% for respectively AP and vertical direction for the hybrid model). Additionally, IMUs can be favourable over pressure insoles, as the insoles may influence the natural interface between the feet and the shoe. Model performance in vertical direction for this study (RMSE of 0.18–0.19BW) is better or comparable with previously reported models. For example, other studies found RMSE of 0.28BW (35), 0.27BW (21), 0.21BW (36), 0.19–0.29BW (37) or 0.16BW (22).

Note that it is tricky to compare different studies as there are multiple ways to calculate model performance. Often, the RMSE between the estimated and measured force is calculated over the stance phase, but not all studies do this (e.g., (19)). Also, the way how the RMSE is calculated can differ, a lower RMSE can be achieved if the RMSE calculated over the flight phase as well. The flight phase is easy to predict as the 3D GRF equals 0N. This reduces the RMSE compared to the method where only data from stance phase is used. It can reduce the RMSE by 0.02BW or rRMSE by 1.2% in vertical direction (See supplementary material). Note that Alcantara et al. also used flight phase data in their calculation (22). Furthermore, it is important to keep in mind that RMSE is a relative measure of model performance, and it depends on the scale of the values. This is reflected by lower values in RMSE for the ML direction compared the vertical direction, while the estimate of the vertical direction is better if we look at the correlation coefficient or rRMSE (Table 1).

The ensemble models outperformed the best ensemble-member for the majority of the GRF estimates (Figure 6), this is in accordance with another study that uses ensemble models in biomechanics (29). In an ensemble model, different subjects were used for training and validation for each ensemble-member. Each ensemble-member is trained with four subjects for validation and the remaining for training. Although there is variation between the validation subjects, it is still expected that the trained ensemble-member has a bias towards the subjects in the validation set. This is supported by the fact that there is a variability in the performance of the ensemble-members (as seen in the range in Figure 6). The bias towards the validation subjects is resolved by combining the different ensemble-members into the ensemble model, improving the generalizability and thus, performance. Although it comes with the cost of computation power, it is recommended to use this approach.

There are multiple ways to ensemble a model (28). In the current study, the average between all members was taken, but other approaches can be used. For example, the weights of different members can be altered based on model performance during training (38). Additionally, model certainty can be derived from the ensemble-members based on the agreement between the members. Predicted GRF waveforms could be discarded if the model confidence is too low. In practice, this can be used with outdoors running; when a runner takes a sharp corner or jumps over a tree root, it can lead to a deviation in the sensor acceleration. This deviation is probably handled differently by the ensemble-members, causing a low agreement and, consequently a low certainty of the estimation.

The hybrid model benefits from the input of the vertical eGRF from the physical model, creating a significantly better estimate in vertical direction than the direct model. Important to note is that the parameters of the physical model are estimated using a part of the data that was used in this study to train the machine learning models. This could mean that the parameters optimized in the physical model are optimized on the current dataset; however, it was shown in a sensitivity analyses that changing model parameters did not had a large effect on the GRF estimation (18). This means that the used physical model is suitable for heel-strikers. In this study, the hybrid model only used vertical eGRF as additional input besides the sensor accelerations; however, more than one physical estimate could be included as input to improve the results. Another method to combine physical and machine learning models would be by using the physical estimate as an ensemble-member.

**Figure 7:**
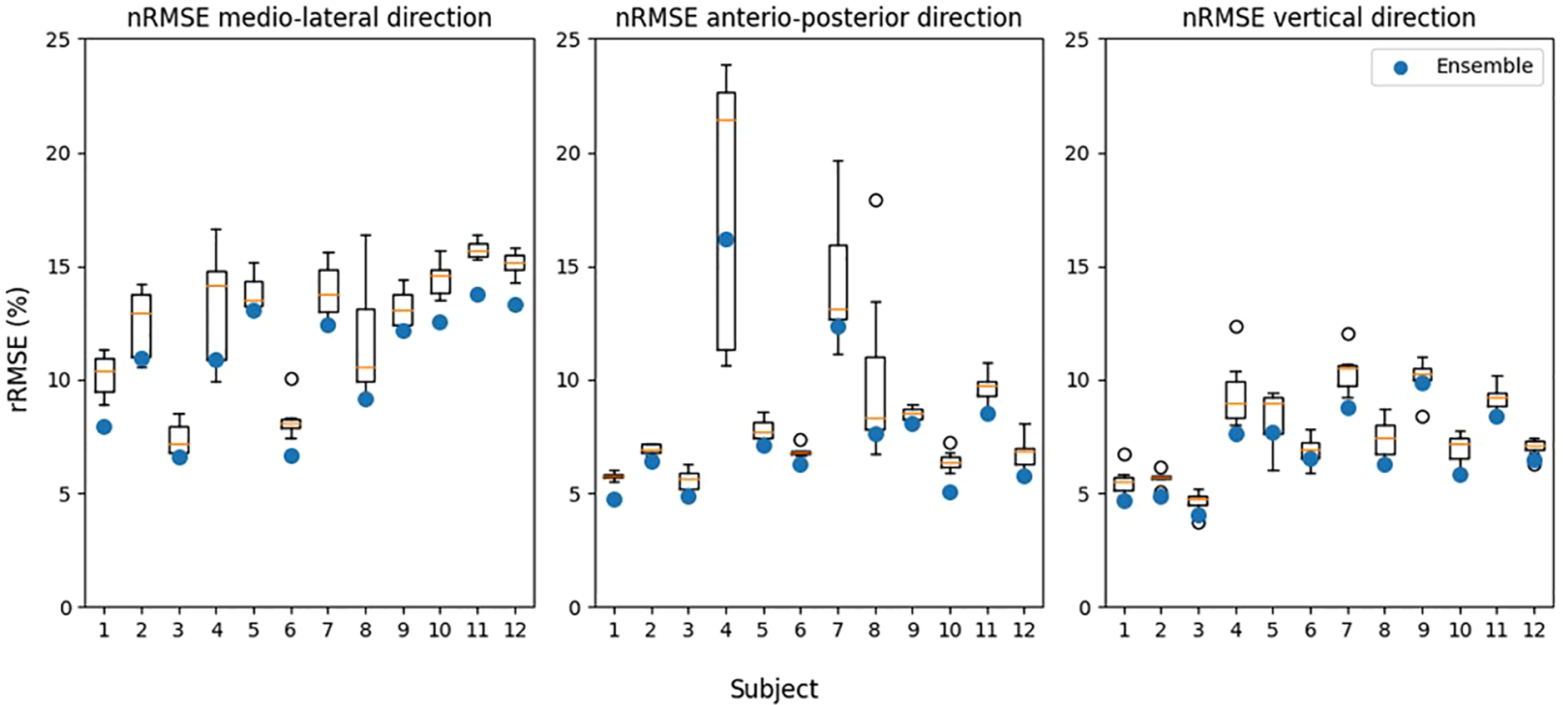
Comparison between performance of the hybrid models and ensemble models with relative root mean squared error (rRMSE) as performance metric. Performance of the single models is shown as the boxplot, the box indicates the lower to upper quartile values of the data, with an orange line at the median. The whiskers show the range of the values, with the black circles as outliers. The ensemble models are indicated with a blue circle. Models were fitted using a leave-one-subject-out cross-validation structure. The average, range and ensemble values can be found in Supplementary material Table S3.

Although there are differences in performance metrics between the various models, the only significant difference found is in the vertical direction for the direct and hybrid model for RMSE, rRMSE and Pearons' r and between the direct and physical model in vertical direction for Pearson's r. This can be explained by the high between-subject variability (Figures 3, 4, 7). For the found significant difference between the direct and hybrid models, the hybrid model outperforms the direct model for 10 out of 12 subjects (based on RMSE). Although the average RMSE in vertical direction is lower for the physical model (0.18BW) compared to the direct model (0.19BW), the direct model still outperforms the physical model for 4/12 subjects.

One important consideration in evaluating the performance of a model for predicting ground reaction forces in running is its clinical and practical applications. While a high degree of accuracy and precision may be desirable in certain situations, such as for research purposes, it may not always be necessary or even desirable in real-world clinical or practical settings. For example, a model with slightly lower accuracy may be more useful if it is faster to compute or easier to implement, or less obtrusive for the user, making it more practical for use in a busy clinical or personal setting. Additionally, the specific requirements of the clinical or practical application should be taken into account when evaluating the model's performance, as a model that is highly accurate and precise for one application may not necessarily be well-suited for another. Also, it is seen that the latest studies (22, 23) are very similar in accuracy. Although there is room to improve these models, it might be more relevant to validate and apply the models in the runners environment.

This study focussed on heel strike runners. Even though the majority of runners has a heel strike [>88% of runners in a competitive road race with 936 runners (39)] it is still not applicable to all runners. Another foot strike pattern is related to another GRF waveform pattern (40), thus the model should still be validated for other foot strike patterns. Another limitation in this study is the relatively low amount of participants. To mitigate this effect, the collected data in this study was made more diverse by imposing different velocities and stride frequencies. As data is time consuming to collect and requires very specific equipment, data augmentation can be used to enrich the data to get more out of the existing data. Data augmentation involves generating additional data points from the original data set through various techniques, such as adding noise, warping, or scaling the data (41).

The sensor attachment is a point of attention when bridging the gap to the practice setting. The current study uses tape fixate the sensors firmly to the body. However, it is not desirable for daily use to tape the sensors to the body. Alternatives could be to use straps, attach sensors clothing [e.g., commercial available Garmin running pod (42)] or even integrate sensors in clothing [as done for a football short (43)]. However, the type of attachment does influence the accelerometer signals (44, 45) and thus the results of the GRF prediction. The method for sensor attachment can also influence the exact sensor location, which also influences the data (46). Future research should investigate the effect of sensor location and attachment on the estimation of GRF. Repeatability of the results when subjects place the sensors themselves should be a point of attention.

Even though there are numerous studies on estimating GRF in the lab setting, there is only one study that used eGRF peak during outside running longitudinally (47), highlighting the gap to the practical setting. As there are differences in kinematics between outdoors and treadmill running (13), it is unclear how well the developed models translate to outdoors circumstances. Probably, the small changes in kinematics between indoor and outdoor running might result in changed kinetics. However, as the models are trained on data from different subjects, with all unique kinetics and kinematics, it is likely that the models account for the variability between in and outdoor running as well. The gap to the practical setting is also identified in a scoping review of IMU based running gait analyses, suggesting that future studies should move out of the lab to a less controlled and more real world-setting to investigate how much the past lab-based studies can be translated into the outdoor setting with real-world conditions such as variations in velocity or road surface (48).

## 5. Conclusion

The main goal of this study was to estimate ground reaction force in running in 3D using data from three IMUs. This study shows that it is possible to estimate GRF in the vertical and anterior-posterior direction for heel strike runners (Pearson's *r* > 0.90). A hybrid model, where data from a physical model is combined with an artificial neural network, showed the best performance for the mediolateral and anterior-posterior direction, however, it was not significantly better than the direct model. For the vertical direction, the physical model had the slightly better, but not significantly better performance. Assembling multiple models into an ensemble increases model performance. A future application of this model is to quantify biomechanical load in the real-world environment, to gain insights in the development of running injuries.

## Data Availability

The raw data supporting the conclusions of this article will be made available by the authors, without undue reservation.

## References

[1] Lee DC Pate RR Lavie CJ Sui X Church TS Blair SN. Leisure-time running reduces all-cause and cardiovascular mortality risk. J Am Coll Cardiol. (2014) 64:472–81. 10.1016/j.jacc.2014.04.058 25082581 PMC4131752

[2] Van Gent RN Siem D Van Middelkoop M Van Os AG Bierma-Zeinstra SMA Koes BW. Incidence and determinants of lower extremity running injuries in long distance runners: a systematic review. Br J Sports Med. (2007) 41:469–80. 10.1136/bjsm.2006.033548 17473005 PMC2465455

[3] Nielsen RO Rønnow L Rasmussen S Lind M. A prospective study on time to recovery in 254 injured novice runners. PLoS One. (2014) 9:e99877. 10.1371/JOURNAL.PONE.0099877 24923269 PMC4055729

[4] Menheere D Janssen M Funk M van der Spek E Lallemand C Vos S. Runner's perceptions of reasons to quit running: influence of gender, age and running-related characteristics. Int J Environ Res Public Health. (2020) 17:1–12. 10.3390/ijerph17176046 PMC7503581 32825266

[5] Fokkema T Hartgens F Kluitenberg B Verhagen E Backx FJG van der Worp H Reasons and predictors of discontinuation of running after a running program for novice runners. J Sci Med Sport. (2019) 22:106–11. 10.1016/j.jsams.2018.06.003 29934211

[6] Yeung SS Yeung EW Gillespie LD. Interventions for preventing lower limb soft-tissue running injuries. Cochrane Database Syst Rev. (2011) 7. 10.1002/14651858.cd001256.pub2 21735382 PMC13283770

[7] Kalkhoven JT Watsford ML Impellizzeri FM. A conceptual model and detailed framework for stress-related, strain-related, and overuse athletic injury. J Sci Med Sport. (2020) 23:726–34. 10.1016/j.jsams.2020.02.002 32111566

[8] Bertelsen ML Hulme A Petersen J Brund RK Sørensen H Finch CF A framework for the etiology of running-related injuries. Scand J Med Sci Sport. (2017) 27:1170–80. 10.1111/sms.12883 28329441

[9] Paquette MR Napier C Willy RW Stellingwerff T. Moving beyond weekly “distance”: optimizing quantification of training load in runners. J Orthop Sport Phys Ther. (2020) 50:564–9. 10.2519/jospt.2020.9533 32741325

[10] Scott SH Winter DA. Internal forces of chronic running injury sites. Med Sci Sports Exerc. (1990) 22:357–69. Available at: http://www.ncbi.nlm.nih.gov/pubmed/2381304 10.1249/00005768-199006000-00013 2381304

[11] Verheul J Nedergaard NJ Vanrenterghem J Robinson MA. Measuring biomechanical loads in team sports–from lab to field. Sci Med Footb. (2020) 4:246–52. 10.1080/24733938.2019.1709654

[12] Healey LA Hoogkamer W. Longitudinal bending stiffness does not affect running economy in nike vaporfly shoes. J Sport Heal Sci. (2022) 11:285–92. 10.1016/j.jshs.2021.07.002 PMC9189697 34280602

[13] Nigg BM De Boer RW Fisher V. A kinematic comparison of overground and treadmill running. Med Sci Sports Exerc. (1995) 27:98–105. 10.1016/S0268-0033(98)00012-6 7898346

[14] Reenalda J Maartens E Homan L Buurke JH. Continuous three dimensional analysis of running mechanics during a marathon by means of inertial magnetic measurement units to objectify changes in running mechanics. J Biomech. (2016) 49:3362–7. 10.1016/j.jbiomech.2016.08.032 27616268

[15] Neugebauer JM Hawkins DA Beckett L. Estimating youth locomotion ground reaction forces using an accelerometer-based activity monitor. PLoS One. (2012) 7:e48182. 10.1371/JOURNAL.PONE.0048182 23133564 PMC3485031

[16] Charry E Hu W Umer M Ronchi A Taylor S. Proceedings of the 2013 IEEE 8th international conference on intelligent sensors, sensor networks and information processing: sensing the future, ISSNIP. Study on estimation of peak ground reaction forces using tibial accelerations in running (2013). p. 288–93. 10.1109/ISSNIP.2013.6529804

[17] Wundersitz DWT Netto KJ Aisbett B Gastin PB. Validity of an upper-body-mounted accelerometer to measure peak vertical and resultant force during running and change-of-direction tasks. Sports Biomech. (2013) 12:403–12. 10.1080/14763141.2013.811284 24466652

[18] Scheltinga BL Usta H Reenalda J Buurke JH. Estimating vertical ground reaction force during running with 3 inertial measurement units. J Biomed Eng Biosci. (2022) 9:31–8. 10.11159/jbeb.2022.006

[19] LeBlanc B Hernandez EM McGinnis RS Gurchiek RD. Continuous estimation of ground reaction force during long distance running within a fatigue monitoring framework: a kalman filter-based model-data fusion approach. J Biomech. (2021) 115:110130. 10.1016/j.jbiomech.2020.110130 33257007

[20] Gurchiek RD Cheney N McGinnis RS. Estimating biomechanical time-series with wearable sensors: a systematic review of machine learning techniques. Sensors (Switzerland). (2019) 19:5227. 10.3390/s19235227 PMC6928851 31795151

[21] Wouda FJ Giuberti M Bellusci G Maartens E Reenalda J van Beijnum BJF Estimation of vertical ground reaction forces and sagittal knee kinematics during running using three inertial sensors. Front Physiol. (2018) 9:1–14. 10.3389/fphys.2018.00218 29623042 PMC5874328

[22] Alcantara RS Edwards WB Millet GY Grabowski AM. Predicting continuous ground reaction forces from accelerometers during uphill and downhill running: a recurrent neural network solution. PeerJ Comput Sci. (2022) 10:e12752. 10.7717/PEERJ.12752 PMC8740512 35036107

[23] Honert EC Hoitz F Blades S Nigg SR Nigg BM. Estimating running ground reaction forces from plantar pressure during graded running. Sensors. (2022) 22(9):3338. 10.3390/s22093338 35591027 PMC9105722

[24] Refai MIM van Beijnum BJF Buurke JH Veltink PH. Portable gait lab: estimating over-ground 3D ground reaction forces using only a pelvis IMU. Sensors. (2020) 20:6363. 10.3390/S20216363 33171858 PMC7664647

[25] Lim H Kim B Park S. Prediction of lower limb kinetics and kinematics during walking by a single IMU on the lower back using machine learning. Sensors (Switzerland). (2020) 20:130. 10.3390/s20010130 PMC6982819 31878224

[26] Karatsidis A Bellusci G Schepers HM de Zee M Andersen MS Veltink PH. Estimation of ground reaction forces and moments during gait using only inertial motion capture. Sensors (Switzerland). (2017) 17:75. 10.3390/s17010075 PMC5298648 28042857

[27] Gurchiek RD Donahue N Fiorentino NM Mcginnis RS. Wearables-Only analysis of muscle and joint mechanics: an EMG-driven approach. IEEE Trans Biomed Eng. (2022) 69:580–9. 10.1109/TBME.2021.3102009 34351852 PMC8820126

[28] Dietterich TG. Ensemble methods in machine learning. Lect Notes Comput Sci (Including Subser Lect Notes Artif Intell Lect Notes Bioinformatics). (2000):1857. LNCS:1–15. 10.1007/3-540-45014-9_1

[29] Grzesiak E Sloboda J Siu HC. Predicting ankle moment trajectory with adaptive weighted ensemble of LSTM networks. in Ieeexplore Ieee org. (2022):1–7. 10.1109/hpec55821.2022.9926370

[30] Machalek D Quah T Powell KM. A novel implicit hybrid machine learning model and its application for reinforcement learning. Comput Chem Eng. (2021) 155:107496. 10.1016/J.COMPCHEMENG.2021.107496

[31] Kingma DP Ba JL. 3rd International conference on learning representations, ICLR 2015—conference track proceedings (international conference on learning representations, ICLR). Adam: a method for stochastic optimization

[32] Abadi M Agarwal A Barham P Brevdo E Chen Z Citro C Tensorflow: large-scale machine learning on heterogeneous distributed systems. arXiv Prepr ArXiv. (2016):160304467. 10.5281/zenodo.4724125

[33] Saeb S Lonini L Jayaraman A Mohr DC Kording KP. The need to approximate the use-case in clinical machine learning. Gigascience. (2017) 6:1–9. 10.1093/GIGASCIENCE/GIX019 PMC5441397 28327985

[34] Gurchiek RD McGinnis RS Needle AR McBride JM van Werkhoven H. The use of a single inertial sensor to estimate 3-dimensional ground reaction force during accelerative running tasks. J Biomech. (2017) 61:263–8. 10.1016/J.JBIOMECH.2017.07.035 28830590

[35] Ohtaki Y Sagawa K Inooka H. A method for gait analysis in a daily living environment by body-mounted instruments. JSME Int J Ser C. (2001) 44:1125–32. 10.1299/jsmec.44.1125

[36] Dorschky E Nitschke M Martindale CF van den Bogert AJ Koelewijn AD Eskofier BM. CNN-Based Estimation of sagittal plane walking and running biomechanics from measured and simulated inertial sensor data. Front Bioeng Biotechnol. (2020) 8:604. 10.3389/FBIOE.2020.00604 32671032 PMC7333079

[37] Donahue SR Hahn ME. Estimation of ground reaction force waveforms during fixed pace running outside the laboratory. Front Sport Act Living. (2023) 5:15. 10.3389/FSPOR.2023.974186 PMC9968876 36860734

[38] Maqsood I Khan MR Abraham A. An ensemble of neural networks for weather forecasting. Neural Comput Appl. (2004) 13:112–22. 10.1007/S00521-004-0413-4

[39] Larson P Higgins E Kaminski J Decker T Preble J Lyons D Foot strike patterns of recreational and sub-elite runners in a long-distance road race. J. Sports Sci. (2011) 29:1665–73. 10.1080/02640414.2011.610347 22092253

[40] Gruber AH Umberger BR Miller RH Hamill J. Muscle mechanics and energy expenditure of the triceps surae during rearfoot and forefoot running. bioRxiv. (2018):424853. 10.1101/424853

[41] Liu B Zhang Z Cui R. Efficient time series augmentation methods. Proc—2020 13th int congr image signal process biomed eng informatics. CISP-BMEI. (2020):1004–1009. 10.1109/CISP-BMEI51763.2020.9263602

[42] Garmin. Running Dynamics. Available at: https://www.garmin.com/en-US/p/561205 (Accessed December 23, 2022)

[43] Steijlen A Burgers B Wilmes E Bastemeijer J Bastiaansen B French P Smart sensor tights: movement tracking of the lower limbs in football. Wearable Technol. (2021) 2. 10.1017/wtc.2021.16 PMC10936253 38486627

[44] Sheerin KR Reid D Besier TF. The measurement of tibial acceleration in runners—a review of the factors that can affect tibial acceleration during running and evidence-based guidelines for its use. Gait Posture. (2019) 67:12–24. 10.1016/j.gaitpost.2018.09.017 30248663

[45] Forner-Cordero A Mateu-Arce M Forner-Cordero I Alcántara E Moreno JC Pons JL. Study of the motion artefacts of skin-mounted inertial sensors under different attachment conditions. Physiol Meas. (2008) 29:N21. 10.1088/0967-3334/29/4/N01 18401071

[46] Lucas-Cuevas AG Encarnación-Martínez A Camacho-García A Llana-Belloch S Pérez-Soriano P. The location of the tibial accelerometer does influence impact acceleration parameters during running. J. Sports Sci. (2017) 35:1734–8. 10.1080/02640414.2016.1235792 27690754

[47] Kiernan D Hawkins DA Manoukian MAC McKallip M Oelsner L Caskey CF Accelerometer-based prediction of running injury in national collegiate athletic association track athletes. J Biomech. (2018) 73:201–9. 10.1016/j.jbiomech.2018.04.001 29699823 PMC6561647

[48] Benson LC Räisänen AM Clermont CA Ferber R. Is this the real life, or is this just laboratory? A scoping review of IMU-based running gait analysis. Sensors. (2022) 22:1722. 10.3390/S22051722 35270869 PMC8915128

