# Supplementary material for: Estimating 3D ground reaction forces in running using three inertial measurement units
Source: Front Sports Act Living. 2023 May 15;5:1176466. doi: 10.3389/fspor.2023.1176466 (PMC10225635; doi:10.3389/fspor.2023.1176466)
Supplement: Supplementary file 1 [file Datasheet1.docx]

Supplementary Material

Assemble your models: estimating 3D ground reaction forces in running using three Inertial Measurement Units

Bouke L. Scheltinga^1*^, Joost N. Kok^3*^, Jaap H. Buurke^1,2^, Jasper Reenalda^1,2^

^1^Biomedical Signals and Systems, Faculty of Electrical Engineering, Mathematics and Computer Science (EEMCS), University of Twente, Enschede, The Netherlands

^2^Roessingh Research and Development, Enschede, The Netherlands

^3^Faculty of Electrical Engineering, Mathematics and Computer Science (EEMCS), University of Twente, Enschede, The Netherlands

*** Correspondence:** Corresponding Author: [b.l.scheltinga@utwente.nl](mailto:b.l.scheltinga@utwente.nl)

Supplementary Table 1: Relative root mean squared error (rRMSE) for each axis per model per subject

| **Direction** | **Mediolateral** | | **Anterior-posterior** | | **Vertical** | | |
| --- | --- | --- | --- | --- | --- | --- | --- |
|  | Direct | Hybrid | Direct | Hybrid | Direct | Hybrid | Physical |
| 01 | 8.3 | 7.9 | 4.9 | 4.8 | 5.0 | 4.7 | 6.1 |
| 02 | 11.1 | 11.0 | 6.5 | 6.4 | 5.5 | 4.9 | 5.2 |
| 03 | 6.7 | 6.6 | 5.0 | 4.9 | 4.1 | 4.0 | 5.3 |
| 04 | 11.6 | 10.9 | 15.8 | 16.2 | 9.1 | 7.6 | 6.3 |
| 05 | 12.7 | 13.1 | 7.6 | 7.1 | 8.5 | 7.7 | 6.4 |
| 06 | 6.9 | 6.7 | 6.6 | 6.3 | 6.1 | 6.6 | 7.7 |
| 07 | 12.2 | 12.4 | 12.7 | 12.4 | 8.5 | 8.8 | 5.2 |
| 08 | 8.7 | 9.1 | 7.3 | 7.6 | 7.2 | 6.3 | 7.0 |
| 09 | 12.2 | 12.2 | 8.4 | 8.1 | 10.5 | 9.9 | 8.7 |
| 10 | 12.9 | 12.6 | 5.6 | 5.1 | 7.5 | 5.8 | 6.6 |
| 11 | 14.2 | 13.8 | 7.9 | 8.5 | 8.6 | 8.4 | 8.7 |
| 12 | 13.5 | 13.3 | 5.9 | 5.8 | 7.1 | 6.5 | 6.3 |

Supplementary Table 2: Pearson’s r for each axis per model per subject

| **Direction** | **Mediolateral** | | **Anterior-posterior** | | **Vertical** | | |
| --- | --- | --- | --- | --- | --- | --- | --- |
|  | Direct | Hybrid | Direct | Hybrid | Direct | Hybrid | Physical |
| 01 | 0.82 | 0.83 | 0.97 | 0.97 | 0.99 | 0.99 | 0.98 |
| 02 | 0.58 | 0.58 | 0.95 | 0.95 | 0.98 | 0.99 | 0.99 |
| 03 | 0.70 | 0.72 | 0.97 | 0.97 | 0.99 | 0.99 | 0.99 |
| 04 | 0.32 | 0.40 | 0.64 | 0.61 | 0.96 | 0.97 | 0.98 |
| 05 | 0.50 | 0.48 | 0.94 | 0.94 | 0.96 | 0.97 | 0.98 |
| 06 | 0.80 | 0.81 | 0.95 | 0.95 | 0.98 | 0.98 | 0.97 |
| 07 | 0.04 | -0.01 | 0.80 | 0.80 | 0.96 | 0.96 | 0.99 |
| 08 | 0.51 | 0.50 | 0.94 | 0.93 | 0.97 | 0.98 | 0.98 |
| 09 | 0.71 | 0.71 | 0.91 | 0.92 | 0.93 | 0.94 | 0.95 |
| 10 | 0.72 | 0.71 | 0.97 | 0.97 | 0.97 | 0.98 | 0.98 |
| 11 | 0.65 | 0.68 | 0.93 | 0.92 | 0.96 | 0.96 | 0.96 |
| 12 | 0.49 | 0.51 | 0.96 | 0.96 | 0.98 | 0.98 | 0.98 |

Supplementary Table 3: Comparison between performance of the hybrid models and ensemble models with relative root mean squared error (rRMSE) as performance metric. Performance of the single models is shown as (mean (range)). Models were fitted using a leave-one-subject-out cross validation structure. The value in the ensemble is bold if it is similar or lower than the lowest ensemble member.

|  | **Mediolateral** | | **Anterior-poster** | | **Vertical** | |
| --- | --- | --- | --- | --- | --- | --- |
| **Subject** | Single (%) | Ensemble (%) | Single (%) | Ensemble (%) | Single (%) | Ensemble (%) |
| 01 | 10.2 (8.9 - 11.3) | **7.9** | 5.8 (5.5 – 6.0) | **4.8** | 5.5 (4.9 - 6.7) | **4.7** |
| 02 | 12.5 (10.6 - 14.2) | 11.0 | 6.9 (6.6 - 7.2) | **6.4** | 5.7 (5.1 - 6.1) | **4.9** |
| 03 | 7.4 (6.5 - 8.5) | 6.6 | 5.6 (5.0 - 6.3) | **4.9** | 4.6 (3.7 - 5.2) | 4.0 |
| 04 | 13.2 (10.0 - 16.6) | 10.9 | 17.7 (10.7 - 23.9) | 16.2 | 9.4 (8.0 - 12.3) | **7.6** |
| 05 | 13.8 (13.0 - 15.2) | 13.1 | 7.8 (7.3 - 8.6) | **7.1** | 8.3 (6.0 - 9.4) | 7.7 |
| 06 | 8.3 (7.4 – 10.0) | **6.7** | 6.8 (6.7 - 7.3) | **6.3** | 6.9 (5.9 - 7.8) | 6.6 |
| 07 | 14 (12.6 - 15.6) | **12.4** | 14.5 (11.2 - 19.6) | 12.4 | 10.4 (9.2 - 12) | **8.8** |
| 08 | 11.7 (9.0 - 16.4) | 9.1 | 10.1 (6.7 - 17.9) | 7.6 | 7.4 (6.4 - 8.7) | **6.3** |
| 09 | 13.1 (12.1 - 14.4) | 12.2 | 8.5 (8.0 - 8.9) | 8.1 | 10.1 (8.4 - 11) | 9.9 |
| 10 | 14.5 (13.5 - 15.7) | **12.5** | 6.4 (5.9 - 7.3) | **5.1** | 7.0 (6.0 - 7.8) | **5.8** |
| 11 | 15.7 (15.3 - 16.4) | **13.7** | 9.6 (8.5 - 10.7) | **8.5** | 9.2 (8.5 - 10.2) | **8.4** |
| 12 | 15.1 (14.3 - 15.8) | **13.3** | 6.7 (5.6 - 8.1) | 5.8 | 7.0 (6.3 - 7.4) | 6.5 |
