# Supplementary material for: Estimating 3D ground reaction forces in running using three inertial measurement units
Source: Front Sports Act Living. 2023 May 15;5:1176466. doi: 10.3389/fspor.2023.1176466 (PMC10225635; doi:10.3389/fspor.2023.1176466)
Supplement: Supplementary file 2 [file Datasheet2.docx]

Supplementary Material

Assemble your models: estimating 3D ground reaction forces in running using three Inertial Measurement Units

Bouke L. Scheltinga^1*^, Joost N. Kok^3*^, Jaap H. Buurke^1,2^, Jasper Reenalda^1,2^

^1^Biomedical Signals and Systems, Faculty of Electrical Engineering, Mathematics and Computer Science (EEMCS), University of Twente, Enschede, The Netherlands

^2^Roessingh Research and Development, Enschede, The Netherlands

^3^Faculty of Electrical Engineering, Mathematics and Computer Science (EEMCS), University of Twente, Enschede, The Netherlands

*** Correspondence:** Corresponding Author: [b.l.scheltinga@utwente.nl](mailto:b.l.scheltinga@utwente.nl)

Model performance calculations

# Introduction

Currently, research to estimate ground reaction forces (GRF) in the runners’ environment is an active research area. Different sensor modalities and model types have been used to estimate GRF in one, two or three dimensions [1]–[4]. However, comparing model performance between different studies is difficult as the metrics for model performance are standardized. Among the commonly reported performance metrics are the root mean squared error (RMSE), relative RMSE (rRMSE) and Pearson correlation coefficient. These measures are calculated over one or multiple strides. However, the outcome of these measures is dependent on whether only stance phase or both stance phase and flight phase data are included. For example, during flight phase, the GRF is 0N by definition, meaning that it is easy to estimate if flight phase can be detected. Calculating the model performance metrics also over flight phase, will thus improve the outcomes. However, it is uncertain how large this effect will be. Therefore, this supplementary material is created to compare the values in the reported error metrics if they are calculated over (1) only stance phase or (2) both stance and flight phase.

# Methods

The hybrid model is used to estimate 3D GRF. This is done by using the leave-one-subject-out cross-validation method. Next, the RMSE, rRMSE and pearson correlation coefficient are calculated for the ensemble model per subject. However, prior to this calculation, there are three different cases (Figure 1):

1. **Stance:** using the force plate data, the stance phase is detected and only data points labelled as stance are used to calculate the performance metrics
2. **Stance + flight:** both data from stance and flight phase is used to calculate the performance metrics
3. **Stance + corrected flight (cflight):** As it is known that the model estimate should predict 0N during flight phase, the estimates labelled as flight are set to 0N. Also, the corresponding measured GRF values are set to 0N.

Then, the performance metrics over the whole population are calculated for each case.


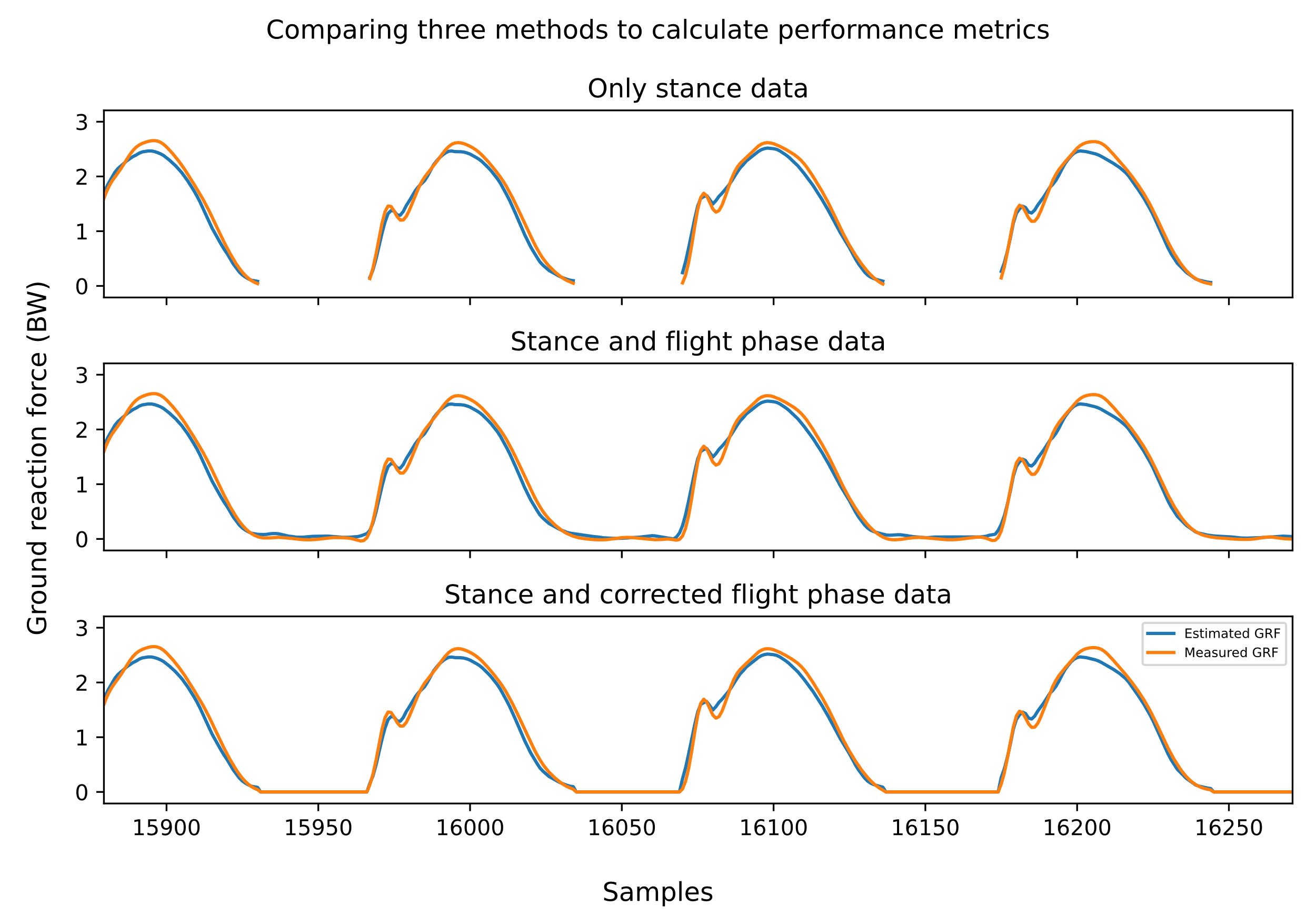


Supplementary Figure 1: Comparison of the three methods how you can calculate performance of the model compared to the measured vertical GRF, with estimated force in blue, measured force in orange. In the top figure, only stance data is included. In the middle, both stance and flight phase data is included. In the bottom, both stance and flight phase data is included, furthermore, the measured and estimated forces are set to 0N if no ground contact was detected. Calculated rRMSE for the shown curves where 4,9, 4,1 and 3,9% seen as from top to bottom.

# Results

A decrease in RMSE and rRMSE is seen for all directions if stance phase is included in the performance calculation (Table 1). Only minor differences are seen in the change in Pearson’s r, with the largest difference in the vertical direction.

Supplementary Table 4: Model performence for the hybrid models with the different methods to calculate the performance metrics. With root mean squared error (RMSE) in bodyweight, relative RMSE (rRMSE) as percentage. The values with stance are the same as Table 1 in the manuscript.

| **Direction** | **Case** | **RMSE (BW)** | **rRMSE (%)** | **Pearson's r** |
| --- | --- | --- | --- | --- |
| Medio-lateral | Stance | 0.05 ± 0.01 | 10.8 ± 2.5 | 0.58 ± 0.22 |
|  | Stance + flight | 0.04 ± 0.01 | 9.4 ± 2.2 | 0.57 ± 0.22 |
|  | Stance + cflight | 0.04 ± 0.01 | 9.1 ± 2.2 | 0.58 ± 0.21 |
| Anterior-posterior | Stance | 0.07 ± 0.03 | 7.8 ± 3.2 | 0.91 ± 0.10 |
|  | Stance + flight | 0.07 ± 0.03 | 6.9 ± 2.6 | 0.90 ± 0.10 |
|  | Stance + cflight | 0.06 ± 0.03 | 6.5 ± 2.7 | 0.91 ± 0.10 |
| Vertical | Stance | 0.18 ± 0.04 | 6.8 ± 1.7 | 0.97 ± 0.01 |
|  | Stance + flight | 0.16 ± 0.04 | 5.6 ± 1.4 | 0.99 ± 0.01 |
|  | Stance + cflight | 0.15 ± 0.03 | 5.6 ± 1.4 | 0.99 ± 0.01 |

# Discussion and conclusion

In the vertical direction, the RMSE decreases with 0.02BW and 1.2% for the rRMSE if the error is calculated over both stance and flight phase, compared to stance phase only. For all directions, lower RMSE and rRMSE are obtained if the flight phase is included in the calculation.

During flight phase, GRFs are 0N by definition. Thus, estimating these forces during flight phase is not relevant. Flight phase can be detected by setting a force threshold on the estimated force or by an specific algorithm [5].

As the model performance is not relevant during flight phase, this data should not be used in the calculation of model performance. Model performance should be calculated only stance phase only, even though this could result in lower model performance.

# References

[1] F. J. Wouda, M. Giuberti, G. Bellusci, E. Maartens, J. Reenalda, B. J. F. van Beijnum, and P. H. Veltink, “Estimation of Vertical Ground Reaction Forces and Sagittal Knee Kinematics During Running Using Three Inertial Sensors,” *Frontiers in Physiology*, vol. 9, pp. 1–14, Mar. 2018, doi: 10.3389/fphys.2018.00218.

[2] R. S. Alcantara, W. B. Edwards, G. Y. Millet, and A. M. Grabowski, “Predicting continuous ground reaction forces from accelerometers during uphill and downhill running: A recurrent neural network solution,” *PeerJ Computer Science*, vol. 10, p. e12752, Jan. 2022, doi: 10.7717/PEERJ.12752/TABLE-3.

[3] G. Vannozzi, A. Merlo, E. C. Honert, F. Hoitz, S. Blades, S. R. Nigg, and B. M. Nigg, “Estimating Running Ground Reaction Forces from Plantar Pressure during Graded Running,” *Sensors 2022, Vol. 22, Page 3338*, vol. 22, no. 9, p. 3338, Apr. 2022, doi: 10.3390/S22093338.

[4] B. L. Scheltinga, H. Usta, J. Reenalda, and J. H. Buurke, “Estimating Vertical Ground Reaction Force during Running with 3 Inertial Measurement Units,” *Journal of Biomedical Engineering and Biosciences*, vol. 9, Jul. 2022, doi: 10.11159/jbeb.2022.006.

[5] L. C. Benson, C. A. Clermont, R. Watari, T. Exley, and R. Ferber, “Automated accelerometer-based gait event detection during multiple running conditions,” *Sensors (Switzerland)*, vol. 19, no. 7, pp. 1–19, 2019, doi: 10.3390/s19071483.
